# Reduced Serum PON1 Lactonase Activity Is Associated with Mild Cognitive Impairment and Mixed Dementia

**DOI:** 10.3390/antiox15081011

**Published:** 2026-08-13

**Authors:** Raffaella Riccetti, Alessandro Trentini, Gianmarco Mola, Gloria Brombo, Dario Francesco di Paola, Massimiliano Castellazzi, Raffaella Candeloro, Maria Cristina Manfrinato, Giovanni Zuliani, Carlo Cervellati

**Affiliations:** 1 Department of Environmental and Prevention Sciences, University of Ferrara, Via Luigi Borsari 46, 44121 Ferrara, Italy; raffaella.riccetti@unife.it; 2 Department of Translational Medicine and for Romagna, University of Ferrara, via Luigi Borsari 46, 44121 Ferrara, Italy; gianmarco.mola@unife.it (G.M.); brmglr@unife.it (G.B.); francescodipao.dario@edu.unife.it (D.F.d.P.); zlngnn@unife.it (G.Z.); 3 Department of Chemical, Pharmaceutical and Agricultural Sciences, University of Ferrara, 44121 Ferrara, Italy; massimiliano.castellazzi@unife.it (M.C.); raffaella.candeloro@unife.it (R.C.); cristina.manfriato@unife.it (M.C.M.)

**Keywords:** PON1, lactonase activity, mild cognitive impairment, dementia

## Abstract

High-density lipoprotein (HDL)-associated paraoxonase-1 (PON1) contributes substantially to the antioxidant and anti-inflammatory functions of HDL. Impairment of these protective properties has been associated with Alzheimer’s disease (AD) and other forms of dementia, particularly those involving neurovascular dysregulation. In the present study, we investigated for the first time the potential involvement of the putative physiological lactonase activity of PON1 in serum samples from a large cohort of older adults (n = 691), including cognitively healthy controls and patients with mild cognitive impairment (MCI), AD, vascular dementia (VAD), mixed AD/VAD (MIXED dementia), and other forms of dementia. A difference was observed between controls and MCI patients (−34%, *p* < 0.001). The next largest differences were observed in the VAD and MIXED dementia groups, both showing a decrease of approximately 25% compared with controls (*p* < 0.05 and *p* < 0.001, respectively), and in AD patients (−14%, *p* < 0.05). After adjustment for confounding factors, only MCI and MIXED dementia remained significantly different from controls. These findings suggest that reduced serum PON1 lactonase activity is already detectable at the MCI stage and is particularly evident in conditions combining neurodegenerative and vascular pathology. Longitudinal studies are required to determine whether lower PON1 lactonase activity precedes, accompanies, or follows cognitive decline.

## 1. Introduction

High density lipoprotein (HDL) represents atheroprotective particles [1]. The mechanisms through which this biological protection is achieved are multiple, ranging from the classically recognized ability to promote cholesterol efflux from macrophages (a key step in reverse cholesterol transport) to more recently acknowledged functions [2,3]. These include antioxidant protection of low-density lipoprotein (LDL) from pro-atherogenic oxidative modification, as well as anti-inflammatory properties, largely mediated through the inhibition of NF-κB activation and downregulation of endothelial adhesion molecules, thereby reducing leukocyte recruitment and vascular inflammation [4].

HDL functions primarily depend on the interplay between its main lipid components, particularly phospholipids [5], and a set of constitutive (mainly ApoA-I) and accessory proteins anchored on the lipoprotein surface [6]. Among this latter highly heterogeneous group, paraoxonase-1 (PON1) has attracted considerable attention over the past decades due to its well-established contribution to HDL’s antioxidant and anti-inflammatory activities [7].

Seminal studies demonstrated the protective role of PON1, highlighting its direct involvement in preventing oxidative modification of lipoproteins and preserving their structural integrity [8]. In particular, Aviram et al. first showed that purified PON1 inhibits copper-induced HDL oxidation [8], a mechanism now recognized as crucial for maintaining both the structural stability and functional properties of HDL [9].

Preclinical and clinical evidence has considerably strengthened interest in PON1 as a potential diagnostic, prognostic, and predictive biomarker, both at the genetic and enzymatic levels, in several disorders, particularly cardiovascular disease, diabetes, and neurodegenerative conditions [10,11,12,13,14,15,16,17]. Overall, the available evidence appears more convincing when PON1 is evaluated through its enzymatic activity rather than through common functional polymorphisms, such as Q192R and L55M, whose associations with disease have often been inconsistent [18,19,20]. On the other hand, an enzymatic approach is itself challenging because of the marked functional pleiotropy of PON1, which is expressed through at least three distinct catalytic activities, paraoxonase, arylesterase, and lactonase, each with different biological and physiological implications, often leading to non-overlapping results in epidemiological and clinical studies [21,22]. The interpretation is further complicated by the ability of common PON1 variants to influence circulating enzyme activity, as its effects on circulating enzymatic activity are strongly substrate-dependent. For instance, the Q192R polymorphism has its most pronounced effect on paraoxon hydrolysis, although possible genotype-dependent differences have also been reported for lactonase activity measured using dihydrocoumarin [23,24]. While paraoxonase and arylesterase activities are generally not considered physiological, as they are measured using exogenous substrates such as organophosphates and arylesters, respectively, lactonase activity is now widely regarded as the most biologically relevant one [25]. Its putative natural substrates include biolactones such as homocysteine thiolactone, as well as a broad range of endogenous lipophilic lactones, including oxidized fatty acid-derived lactones generated in cell membranes and lipoproteins under oxidative stress. [21,22].

Despite the recognized biological relevance of lactonase activity relative to paraoxonase and arylesterase activities, the available literature, particularly in neurodegenerative disorders such as AD, remains limited and does not yet allow firm conclusions to be drawn. The strongest evidence linking PON1 to AD comes from preclinical in vivo studies showing altered PON1 expression in brain regions characterized by abundant amyloid pathology in animal models of the disease [26,27]. Collectively taken, in vivo and in vitro findings suggest that the relationship between PON1 and AD pathology may involve the enzyme’s ability to modulate autophagy. In this regard, Witucki and Jakubowski reported that reduced PON1 expression was associated with lower levels of autophagy markers and increased Aβ accumulation [28]. Human studies provide indirect support for this association, showing reduced serum arylesterase and paraoxonase activities in patients with AD compared with healthy controls, suggesting that alterations in PON1 related to brain pathology may also be reflected at the systemic level [17,29,30,31,32,33,34]. However, studies investigating lactonase activity are still scarce, despite its physiological relevance and its potential association with the alterations observed in AD [30]. Available evidence is limited by small sample sizes and simple case–control designs. To address this gap, this study examined, for the first time to our knowledge, serum lactonase levels in a large sample, comparing cognitively healthy controls against patients with AD, MCI, a condition prodromal to dementia, and other forms of dementia.

## 2. Materials and Methods

**Figure 1:**
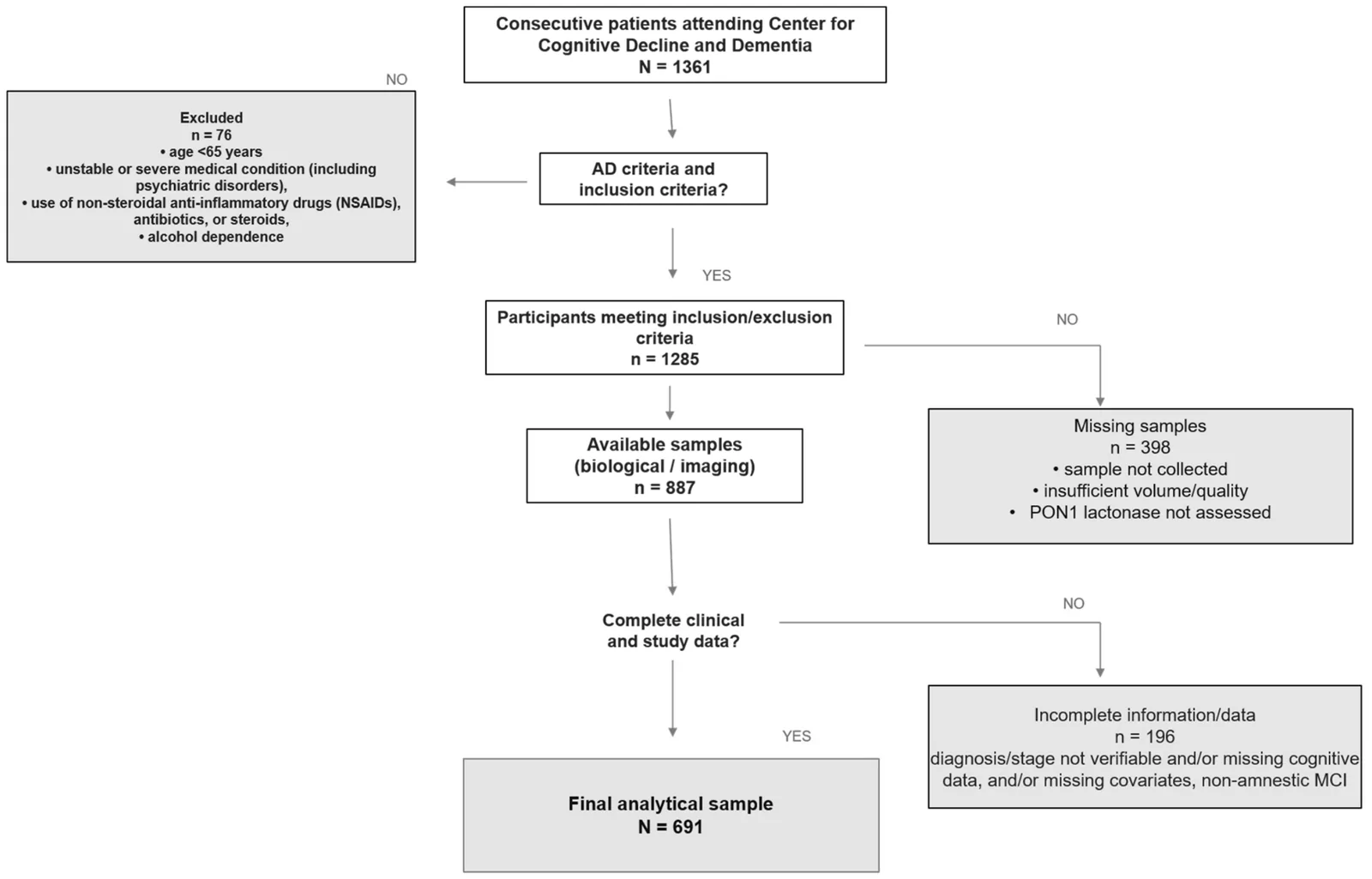
Study flowchart.

A total of 691 elderly patients attending the Day Service for Cognitive Decline at the University of Ferrara were consecutively recruited in this study from 2017 to 2026 (participant selection flow diagram is shown in Figure 1). The study population involved the following groups:

AD (n = 157): Patients with mild-to-moderate Alzheimer’s disease, diagnosed according to the National Institute on Aging–Alzheimer’s Association (NIA-AA) criteria [35,36].

VAD (n = 37): Patients characterized by cognitive decline through neuropsychological testing and detection of cerebrovascular lesions through neuroimaging or clinical stroke history defined according to the National Institute of Neurological Disorders and Stroke–Association Internationale pour la Recherche et l’Enseignement en Neurosciences (NINDS-AIREN) criteria [37].

Mixed AD/VAD (MIXED dementia, n = 199): Patients were assigned to the MIXED dementia group when they presented with a clinical cognitive phenotype compatible with AD, together with cerebrovascular lesions on magnetic resonance imaging (MRI) considered sufficiently severe, extensive, or strategically located to contribute to the cognitive syndrome [38]. Clinical features could include memory impairment and progressive cognitive decline, such as spatial and temporal disorientation, language difficulties, impaired performance of activities of daily living, focal neurological signs, and evidence of significant vascular disease. Accordingly, patients were not classified as having MIXED dementia solely because a definitive diagnosis of either AD or VAD could not be established, but rather on the basis of the coexistence of clinically and radiologically relevant neurodegenerative features and vascular features. MRI findings considered relevant are described later in text.

Other forms of dementia (n = 50): Patients, including those diagnosed with Lewy body disease/Parkinson’s disease dementia, frontotemporal dementia, psychiatric-related cognitive impairment, neoplasm/metastasis, hydrocephalus, Fahr’s syndrome, alcohol-related dementia, post-syphilis, hypothyroidism, and other undefined forms. Because each of these diagnoses was represented by only a small number of individuals, separate subgroup statistical analyses would have been insufficiently powered. Therefore, they were pooled to retain the full clinical spectrum of the recruited cohort at our memory clinic and to allow us an exploratory comparison against the principal diagnostic groups. This grouping was not intended to imply biological or etiological homogeneity among the included conditions.

MCI (n = 139): MCI was diagnosed clinically according to Petersen and the core NIA-AA criteria [39,40]. Diagnosis required: (i) a cognitive concern reported by the patient, an informant, or a clinician; (ii) objective evidence of impairment in one or more cognitive domains; (iii) substantial preservation of independence in activities of daily living; and (iv) absence of dementia. Only patients with amnestic MCI (aMCI) were included to reduce the clinical heterogeneity of the study population. aMCI was defined by objective memory impairment greater than expected for the patient’s age and educational level, with or without impairment in additional cognitive domains. Cognitive impairment primarily attributable to major depression, delirium, severe psychiatric disorders, or other neurological or medical conditions was excluded on the basis of clinical assessment, medical history, and laboratory investigations. One hundred and seven MCI subjects were followed up for a mean period of 21 ± 14 months. During this follow-up period, 61 subjects converted to dementia, whereas 46 remained stable [39].

Controls: Individuals (n = 109) with subjective cognitive complaints but no clinical signs of dementia, no functional impairment attributable to cognitive decline, and normal performance on cognitive testing.

Exclusion criteria were as follows: age  <  65 years; any unstable or severe medical condition (e.g., severe hepatic or renal disease, unstable cardiovascular disease, cancer); dementia due to secondary cases (e.g., B-12 vitamin deficit); major psychiatric disorders such as uncontrolled depression and schizophrenia; and use of NSAIDs, antibiotics, or steroids. Neuropsychological tests were carried out as previously described [41]. At the time of clinical evaluation and blood sampling, no participants showed evidence of acute illness. All subjects underwent comprehensive clinical and neuropsychological assessments, including the Mini-Mental State Examination (MMSE) and 15-item Global Depression Scale (GDS, with a score ≥5 considered indicative of clinically relevant depressive symptoms [42]), as previously described. Functional status was assessed using the Basic Activities of Daily Living (BADL) and Instrumental Activities of Daily Living (IADL) scales. All patients underwent a brain MRI (Philips Achieva 1.5 T) or a brain computed tomography scan (SIEMENS SOMATON HQ) when MRI was contraindicated or the patient’s age was over 85 years. MRI findings considered relevant included leukoaraiosis, assessed using the Fazekas scale; cerebral atrophy, assessed using the Scheltens scale; lacunar infarcts; cortical or strategically located infarcts; and other clinically significant cerebrovascular lesions [38]. Fazekas and Scheltens scores were dichotomized as absent (score = 0) or present (score ≥ 1). Examinations were interpreted by radiologists as part of routine clinical practice. The readers were unaware of the PON1 lactonase results but had access to the relevant clinical information. Diagnoses were established by at least two clinicians with expertise in geriatrics, neuropsychology, and neuroradiology. The diagnostic evaluation was performed before, and independently of, the measurement/availability of the biomarker investigated in the present study; therefore, clinicians were blinded to biomarker values at the time of diagnostic classification. In cases of disagreement, the diagnosis was discussed with an additional experienced clinician or within a multidisciplinary team.

Demographic data and medical history (including hypertension, coronary heart disease, stroke, diabetes, and chronic obstructive pulmonary disease) were collected by trained personnel. Routine laboratory analyses were performed to exclude secondary causes of cognitive impairment, including measurements of vitamin B12 and folate levels, liver, kidney, and thyroid function tests, complete blood count, and arterial oxygen saturation.

Albumin, creatinine, C-reactive protein, hemoglobin, total cholesterol, triglycerides, LDL-cholesterol (LDL-C), and HDL-cholesterol (HDL-C) concentrations were measured by routine laboratory methods.

The study was approved by the Local Ethic Committees (cod number: 170579. This study conforms to The Code of Ethics of the World Medical Association (Declaration of Helsinki, 1975) and was conducted according to guidelines for Good Clinical Practice (European Medicines Agency). Written informed consent was obtained from all participants prior to enrollment, in accordance with local and national ethical regulations.

### 2.1. Assessment of Biomarkers of HDL Function in Serum

Blood samples collected were centrifuged at 1600× *g* for 15 min to isolate serum or plasma. The separated components were then divided into aliquots and frozen at −80 °C for later analysis. All the biochemical parameters were assessed by the spectrofluorometer Tecan Infinite M200 (Tecan Group Ltd., Männedorf, Switzerland).

### 2.2. PON1 Lactonase Activity

The lactonase activity of PON1 was determined through enzymatic hydrolysis of dihydrocoumarin (DHC), a specific artificial substrate [30]. Briefly, 3 µL of serum (diluted 1:5) were added in duplicate to the wells in a UV microplate. Subsequently, each well was treated with 97 µL of Reaction Buffer (50 mM Tris, 1 mM CaCl_2_, pH 7.5) and 100 µL of DHC (Sigma-Aldrich, St. Louis, MO, USA; Cat. No. D104809) at the final concentration of 2 mM. The resulting absorbance was measured at the wavelength of 270 nm following a kinetic cycle every 30 s for 10 min at 37 °C. The absorbance was converted into enzyme Units (U)/L of active enzyme using a molar extinction coefficient ε = 0.0136 µM^−1^ cm^−1^. The assay demonstrated good reproducibility, with an intra-assay coefficient of variation of 5.57% and an inter-assay coefficient of variation of 10.1%.

### 2.3. Oxidized-HDL (oxHDL)

This method is based on a published procedure [43], which uses the enzyme horseradish peroxidase (HRP) and the fluorochrome AmpliFlu Red that can quantify the lipid peroxide content (without cholesterol oxidase) per mg of HDL-C. A quality control from pooled serum of all study samples was set up combining 10 μL from each of them. Then, 40 μL of serum was precipitated with 40 μL of HDL-C precipitating Reagent (20% *w*/*v* polyethylene glycol in 0.1 M glycine buffer at pH 10). All samples were centrifuged for 10 min at 2000× *g* at RT and the supernatant containing the HDL fraction was aspirated. The total amount of cholesterol was quantified using a commercial kit (Cholesterol Quantification Assay kit, Sigma-Aldrich Cat. No. CS0005). Both samples and reagents were prepared in Reaction Buffer 0.5 M KH_2_PO_4_, pH 7.4, 0.25 M NaCl, 25 mM cholic acid, and 0.5% Triton X-100. Subsequently, 50 μL of Reaction Buffer (negative control), pooled control and samples (diluted 1:4) were dispensed in duplicate into the wells of a flat black bottom microplate. Then, 50 μL of 5 U/mL HRP (Sigma-Aldrich, Cat. No. P8375) was added in each well and the plate was incubated for 30 min at 37 °C. Finally, 0.3 mM AmpliFlu Red (Sigma-Aldrich, St. Louis, MO, USA, Cat. No. 90101) was added and the resulting fluorescence was measured at an excitation wavelength of 530 nm and an emission wavelength of 590 nm every minute for 1 h at 37 °C. The mean value of fluorescence units (arbitrary units) was subtracted from the blank by using the following equation:

Fluorescence in HDL sample (FU) − Fluorescence in blank (no HDL) (FU) = oxHDL_sample.

The resulting value of both samples and pool was then normalized for Total Cholesterol amount (mg/dL) by using the following equation:

oxHDL _sample/Tot.Cholesterol mg/dL= oxHDL _normalized

The resulting value of each sample was standardized against the value of the pooled control by using the following equation:

oxHDL _normalized_samples/oxHDL _normalized_pool = nHDLox

The assay showed an intra- and inter-assay, with coefficients of variation 1.94% and 3.93%, respectively.

### 2.4. Myeloperoxidase (MPO) Activity

MPO activity was measured using an immunocapture-based fluorimetric assay as previously published [44]. Briefly, Nunc-Maxisorp ELISA plates with C-shaped wells (hermo Fisher Scientific, Waltham, MA, USA, Cat. No. 446612) were coated overnight at 4 °C with 100 µL/well of polyclonal anti-MPO antibody (Calbiochem, Merck, Darmstadt, Germany, Cat. No. 475915), diluted 1:500 in 0.2 M sodium bicarbonate buffer, pH 9.4. Plates were then washed three times with 300 µL/well of wash buffer (WB: 0.15 M NaCl, 0.1 M NaH_2_PO_4_, pH 7.2, containing 0.05% Tween-20). Non-specific binding sites were blocked with 300 µL/well of 5% BSA in WB for 1 h at room temperature under gentle shaking.

After three additional washes with WB, 100 µL of serum samples (diluted 10 times) or MPO standards (leukocyte-derived MPO, Calbiochem, Merck, Darmstadt, Germany, Cat. No. 475911, at concentrations ranging from 0.39 to 25 ng/mL) were added to the wells in duplicate. Samples were diluted in 1% BSA prepared in WB without Tween-20, used as dilution buffer. Following incubation for 1 h at room temperature with gentle agitation, plates were washed four times with WB.

The enzymatic reaction was started by adding 50 µL of 392 µM H_2_O_2_ and 50 µL of 200 µM AmpliFlu Red reagent (Sigma-Aldrich, Cat. No. 90101) to each well. Both reagents were prepared in 20 mM citrate buffer, pH 6.0 and supplemented with 80 mM NaBr, resulting in final assay concentrations of 196 µM H_2_O_2_ and 100 µM AmpliFlu Red. AmpliFlu Red was prepared from a 200 mM stock solution in DMSO and stored in aliquots at −20 °C. The product (resorufin) fluorescence was monitored at 37 °C for 10 min, with readings every 30 s, using excitation and emission wavelengths of 535 and 590 nm, respectively, on a Tecan Infinite M200 microplate fluorimeter (Tecan Group Ltd., Männedorf, Switzerland).

Fluorescence values were converted into U/mL of active MPO using a resorufin-based standard curve, as previously described [44]. Assay validation showed performance comparable to previous applications, with intra-assay and inter-assay coefficients of variation of 5.5 ± 1.7% and 8.3 ± 2.2%, respectively.

### 2.5. Statistical Analysis

The dataset was checked for completeness before statistical analysis. No missing values were present for the variables included in the primary and subgroup analyses; therefore, no missing data procedure was required.

Prior to statistical analyses, data were screened for outliers using boxplots and standardized z-scores. Values with z-scores greater than ±3 were considered potential outliers. The distribution of continuous variables of interest was assessed using the Kolmogorov–Smirnov and Shapiro–Wilk tests.

Group comparisons were performed using analysis of variance (ANOVA), with Bonferroni post hoc correction for pairwise comparisons, for continuous variables, and the chi-square test for categorical variables. Analysis of covariance (ANCOVA) was used to determine whether differences observed in univariate analyses remained significant after adjustment for potential confounding factors. The selection of covariates for the primary ANCOVA model was based on their established or biologically plausible relationships with circulating PON1 activity and cognitive or vascular diagnosis. Age and sex were included as demographic determinants of PON1 activity; HDL-C was included due to the direct biological relationship with PON1; smoking, diabetes, hypertension, and previous stroke were included as vascular and metabolic factors potentially associated with PON1 activity.

Pearson’s or Spearman’s correlation coefficients were used, as appropriate according to the normality or non-normality of data distribution, to assess associations between variables of interest. Correlation analyses were followed by multiple regression analyses to evaluate the independence of the observed associations.

No global correction was applied across the secondary subgroup, correlation, and neuroimaging analyses, which were considered exploratory and hypothesis-generating. Therefore, *p*-values from these analyses should be interpreted cautiously in view of the increased probability of type I error. No a priori sample-size calculation was performed because the study was based on an existing cohort and available stored biological samples.

A receiver operating characteristic (ROC) curve was performed to evaluate the diagnostic accuracy of the examined biomarker in discriminating controls from cases.

A *p*-value < 0.05 was considered statistically significant. All analyses were performed using SPSS version 17.

## 3. Results

### 3.1. Main Demographic and Clinical Data

Table 1 summarizes the main demographic characteristics, neuropsychiatric and functional test outcomes, comorbidity prevalences, and clinical blood chemistry biomarkers of the study population, including controls and individuals with MCI, AD, VAD, MIXED dementia, and other forms of dementia. The controls was the youngest group, with a significant age difference compared with all patient groups, ranging from 3 to 5 years (*p* < 0.01 for all comparisons), whereas no significant age differences were observed among the patient groups. Women were more prevalent across all groups, with the highest proportions observed in the AD and VAD groups, at approximately 70%.

As expected, MMSE, IADL, and BADL scores were significantly lower in the patient groups than in controls; MMSE scores were also lower in dementia groups than in the MCI group. No significant between-group differences were observed for comorbidities or blood biomarkers, with the sole exception of albumin, which was slightly lower in the MIXED dementia and other forms of dementia groups than in controls (*p* < 0.05).

### 3.2. PON1 Lactonase Across Sample Groups

**Figure 2:**
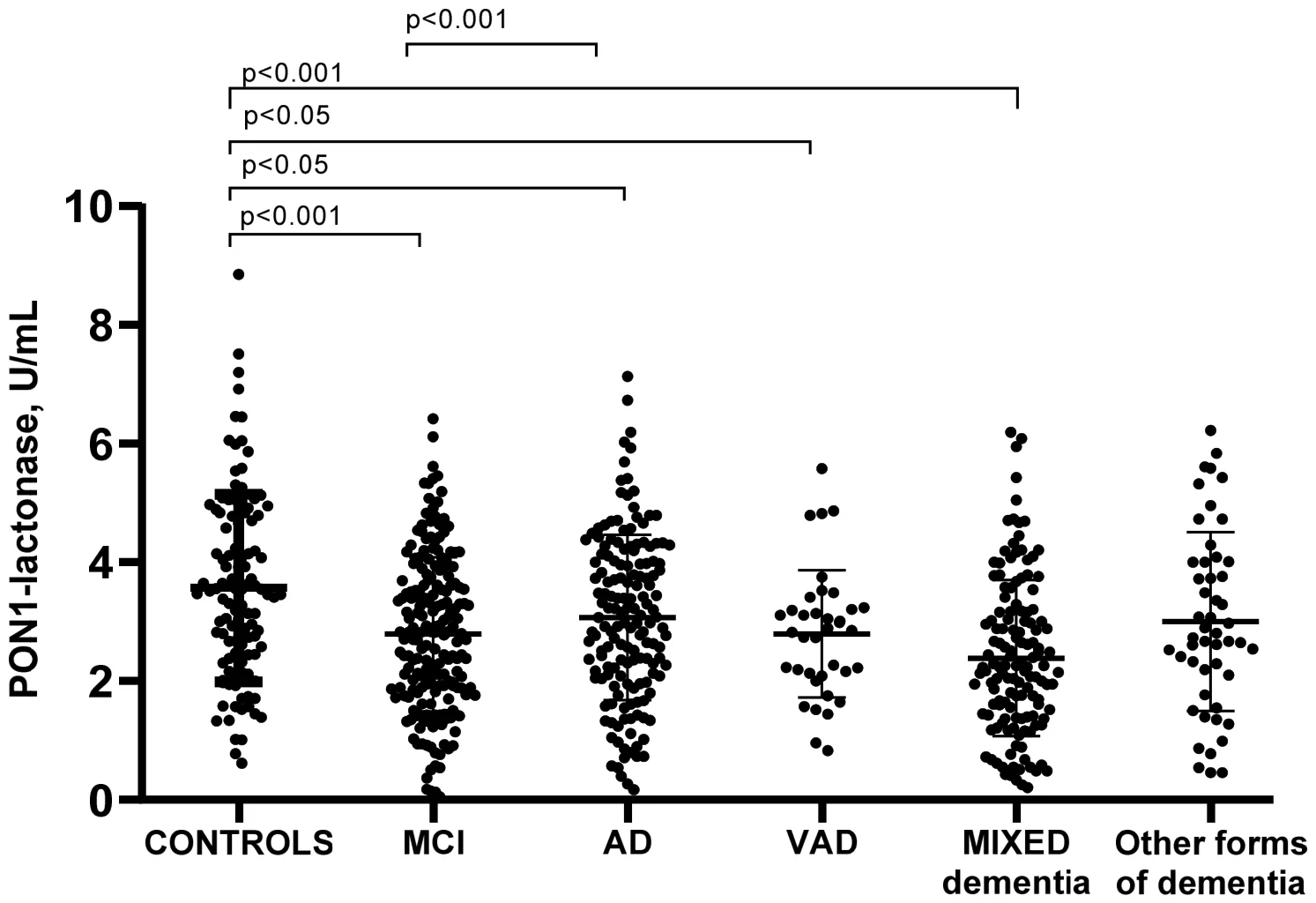
Serum PON1 lactonase activity across the study groups.

Serum PON1 lactonase activity was significantly lower in the MCI and dementia groups than in controls, except for the group with other forms of dementia (Figure 2). More specifically, the largest difference was observed between controls and MCI patients (−34%, *p* < 0.001), followed, in order of magnitude, by VAD and MIXED dementia groups, both showing a decrease of approximately 25% compared with controls (*p* = 0.04 and *p* < 0.001, respectively), and by AD patients (−14%, *p* = 0.05). A highly significant difference was also found between the MCI and AD groups, with PON1 lactonase activity being 22% lower in MCI than in AD.

Serum PON1 lactonase activity was significantly lower in participants with MCI, AD, VAD and MIXED dementia, but not in other forms of dementia, compared to controls.

Notably, we also tested whether PON1 lactonase activity differed between MCI patients who remained stable and those who converted to dementia. Although the difference did not reach statistical significance, converters showed lower PON1 lactonase activity than stable MCI patients (−17%) (Figure S1).

Because controls significantly differed in age from other groups, and because other factors such as sex, comorbidities and HDL-C may influence PON1 activity [45,46], we used ANCOVA to evaluate the potential influence of these factors on the observed differences in this biomarker among the study groups (Table 2).

As shown in Table 2, mean enzyme activity levels remained largely unchanged after multivariable adjustment. However, some group differences were no longer significant, namely controls vs. AD and controls vs. VAD. Other differences remained significant, including controls vs. MCI (*p* < 0.001), controls vs. MIXED dementia (*p* = 0.029), and MCI vs. AD (*p* = 0.002).

Among the covariates included in the multivariable model, age (*p* < 0.01), sex (*p* < 0.05), and HDL-C (*p* < 0.01) were associated with PON1 lactonase activity, whereas smoking status and comorbidities were not. However, these associations did not translate into significant interactions with PON1 lactonase activity.

Diagnostic accuracy of PON1 lactonase activity was evaluated by ROC curves and the area under ROC curves (AUC) calculation (Figure S2), using the best compromises between sensitivity and specificity for the diagnosis of MCI, AD, VAD and MIXED dementia. AUC values were 0.721 (sensitivity/specificity: 73/62%, cut-off = 2.5 U/mL) for MCI, 0.581 (sensitivity/specificity: 61/50%, cut off = 3.0 U/mL,) for AD, 0.641 (sensitivity/specificity: 61/60%, cut off = 2.9 U/mL) for VAD, and 0.659 (sensitivity/specificity: 67/50%, cut off = 2.6 U/mL) for MIXED dementia.

### 3.3. Stratification of PON1 Lactonase Activity by Age, Sex, and HDL-C: Effects on the Relationship Between the Marker and Patient Diagnosis

In agreement with previous investigations based on another PON1 activity, arylesterase, PON1 lactonase activity was 10% higher in women than in men (*p* < 0.05). This difference was mainly driven by controls and AD, where women showed 17 and 14% higher PON1 lactonase activity than men (*p* < 0.05), respectively (Figure S3). By contrast, PON1 lactonase levels remained largely unchanged between sexes in patients with MCI, VAD, MIXED dementia, and other forms of dementia. These sex-related variations were reflected in the statistical analysis of differences across diagnostic groups (Figure S4). In women, PON1 lactonase activity was significantly higher in controls than in patients with MCI (*p* < 0.001) and MIXED dementia (*p* < 0.001), while MCI patients showed significantly lower activity than AD patients (*p* < 0.01). Conversely, in men, the only significant difference retained was between controls and MCI patients (*p* < 0.05).

The effects of age and HDL-C were evaluated by stratifying the study population according to the median values of these variables, namely 79 years for age and 59 mg/dL for HDL-C. Overall, PON1 lactonase activity showed a weak but significant negative correlation with age (r = −0.126, *p* < 0.01) and a positive correlation with HDL-C levels (r = 0.211, *p* < 0.001, ). When comparing younger and older subgroups (Figure S5), PON1 lactonase activity was significantly higher in controls than in MCI patients in both age strata. However, the difference between controls and patients with MIXED dementia was retained only in the younger subgroup. A similar pattern was observed after stratification by HDL-C levels. Both differences were preserved only among subjects with HDL-C levels above the median (Figure S6), whereas in subjects with lower HDL-C levels, only the difference between controls and MCI patients remained significant. Substantially, the difference that appears to be conserved in these stratification analysis appears to be those between controls and both MCI and MIXED dementia.

**Figure 3:**
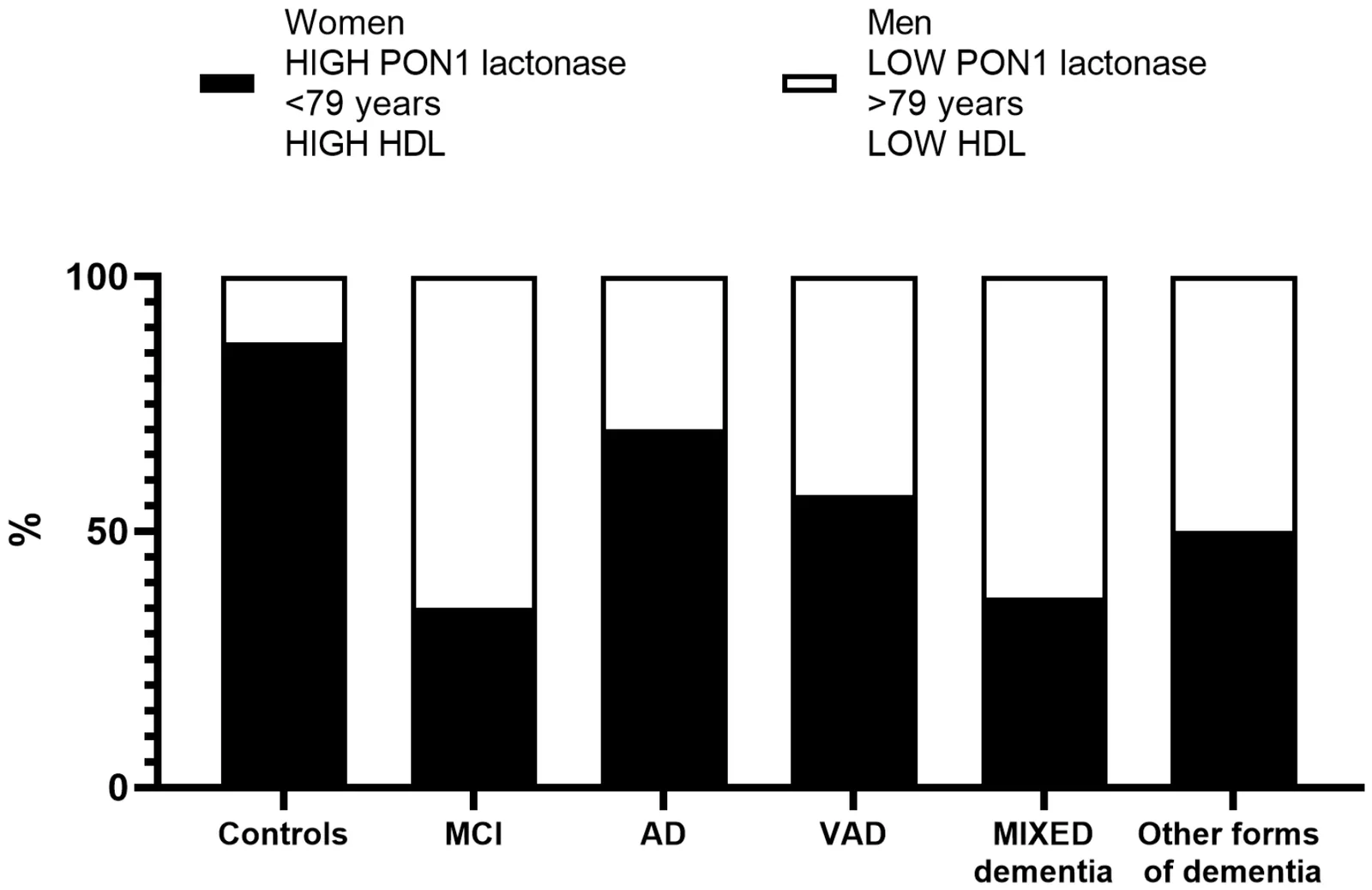
Prevalence of subjects with “healthy” and “unhealthy” profiles across the groups.

In an exploratory analysis, the combination of female sex, age below 79 years, higher HDL-C levels and higher PON1 (median value of 3.5 U/mL was used as cut-off) lactonase activity was more frequent among cognitively healthy controls. Therefore, we can hypothesize that this profile of biomarkers could be related to a more “healthy” congnitive state. However, given the exploratory nature of this analysis, this finding should be interpreted cautiously and requires confirmation in independent cohorts. Visual support for our hypothesis is provided in Figure 3, where this “healthy” combination was clearly more frequent among controls. Indeed, 87% of control subjects showed this profile, whereas it was poorly represented in patients with MCI and MIXED dementia, being present in approximately 35% of subjects in both groups.

The so-called “healthy” profile was present in the large majority of controls, whereas it was poorly represented especially in patients with MCI and MIXED dementia.

### 3.4. Correlation Between PON1 Lactonase Activity and Oxidized-HDL

To assess whether PON1 lactonase activity may protect HDL from oxidation and from the potential pro-oxidant and pro-inflammatory properties of these lipoproteins, correlation analyses were performed between PON1 lactonase activity and both oxHDL levels and MPO activity.

A sufficient number of serum samples was available for controls (n = 68 for oxHDL and n = 25 for MPO activity), MCI patients (n = 33 for both markers), AD patients (n = 111 for oxHDL and n = 57 for MPO activity), and patients with MIXED dementia (n = 76 for both markers).

**Figure 4:**
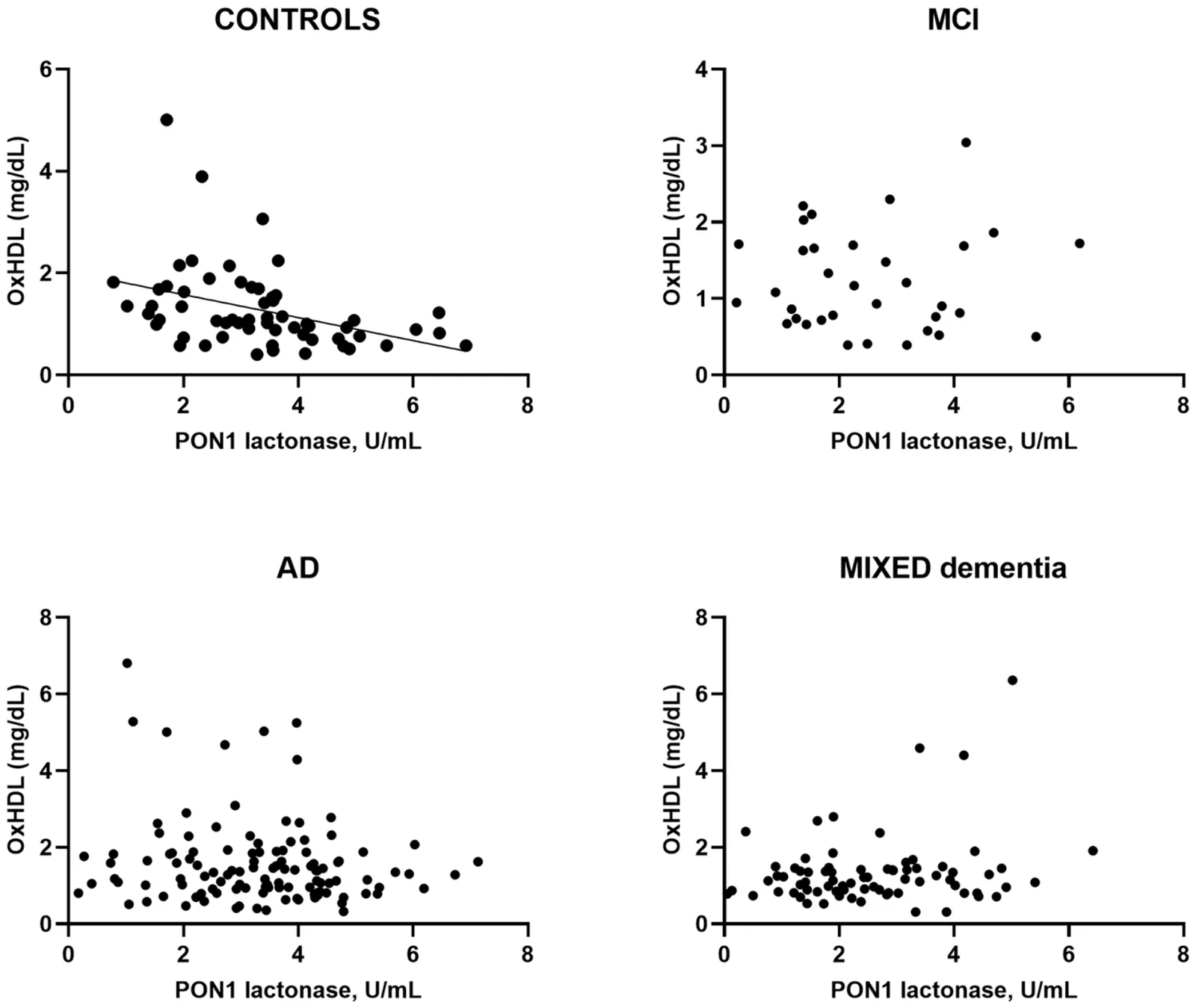
Correlations between Serum PON1 lactonase activity and oxHDL among controls, MCI, AD and MIXED dementia.

In the overall study population, no significant correlations were observed between PON1 lactonase activity and oxHDL levels (r = −0.071), or between PON1 lactonase activity and MPO activity (r = 0.035). However, when the association was examined separately within each diagnostic group, a marked heterogeneity emerged for the relationship between PON1 lactonase activity and oxHDL levels (Figure 4). More specifically, this correlation was significant only among controls, in whom higher PON1 lactonase activity was associated with lower oxHDL levels (r = −0.485, *p* < 0.01).

Serum PON1 lactonase was significantly correlated with oxHDL only in controls.

### 3.5. Association Between PON1 Lactonase Activity, Cognitive and Functional Test Performance, and MRI Outcomes

**Figure 5:**
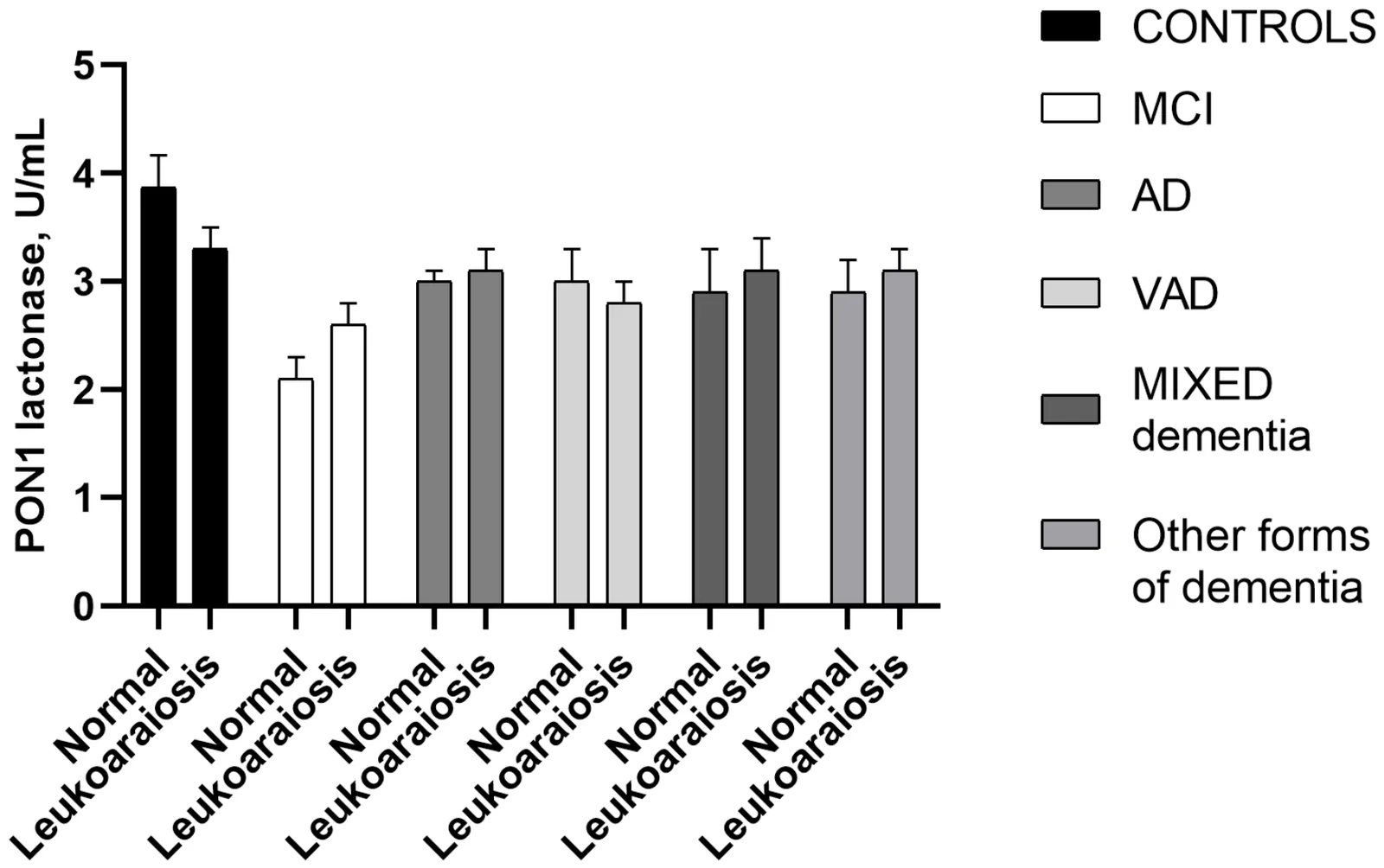
Serum PON1 lactonase activity according to the presence or absence of leukoaraiosis in each diagnostic group.

**Figure 6:**
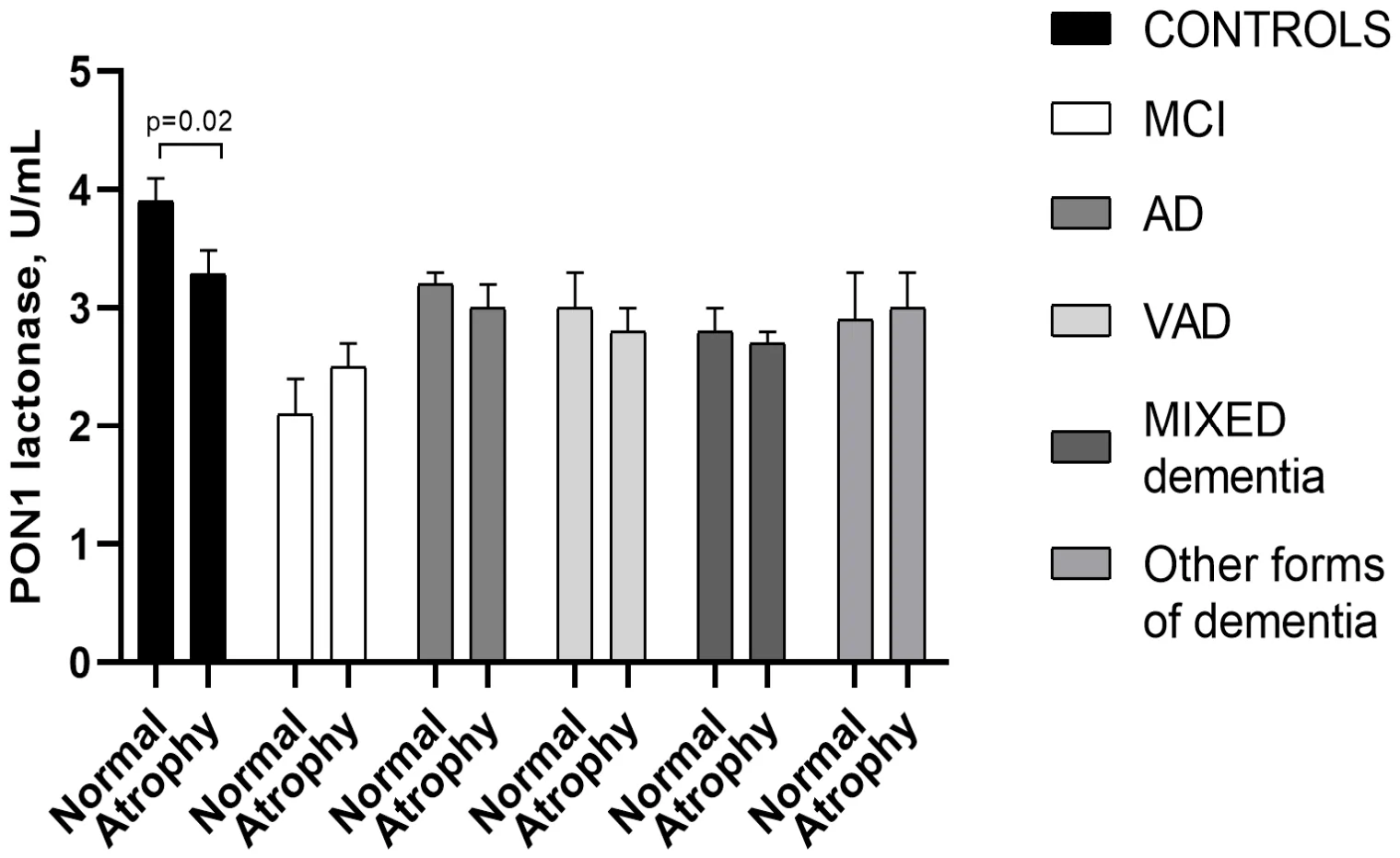
Serum PON1 lactonase activity according to the presence or absence of cerebral atrophy in each diagnostic group.

No significant correlations were found between PON1 lactonase activity and cognitive or functional parameters, including MMSE, IADL, and BADL scores. Considering the whole sample, similar negative results were obtained for MRI outcomes. In exploratory analyses stratified by diagnostic group, significant and biologically meaningful associations were observed among controls (Figure 5 and Figure 6). In this group, PON1 lactonase activity was lower in subjects with leukoaraiosis (−15%, Figure 5) and cerebral atrophy (−18%, *p* < 0.05, Figure 6).

Low Serum PON1 lactonase was associated with the presence of leukoaraiosis mostly in controls, while the difference in the other study groups was limited.

Low Serum PON1 lactonase was significantly associated with the presence of brain atrophy in controls, while the difference in the other study groups was limited.

## 4. Discussion

The possible association between PON1 and disease occurrence, including dementia-related disorders, has been investigated almost exclusively through the measurement of substrate-promiscuous esterase activities, such as arylesterase and paraoxonase activities [17,29,30,31,32,33,34]. Our previous works have mainly focused on arylesterase activity, which is widely considered the best indirect estimate of PON1 concentration [29,47]. These studies showed that arylesterase activity was lower across several diagnostic groups, including individuals with preclinical cognitive alterations, MCI, AD, VAD, and MIXED dementia than in corresponding control groups.

In the present study, we investigated whether the previously reported lower estimates of PON1 concentration were mirrored by lower serum lactonase activity across diagnostic groups. In fact, this activity is thought to be the one most directly involved in the antioxidant function of HDL-associated PON1. Indeed, PON1 lactonase activity may contribute to antioxidant protection by hydrolyzing lipid peroxidation products, particularly lipolactones, thereby limiting the propagation of oxidative damage across lipid layers [25,43,48,49].

We obtained results that were similar to, but did not fully overlap with, those observed for the other PON1 activities. Serum PON1 lactonase activity showed a broadly comparable trend, although the magnitude of the differences varied across diagnostic groups [29,47]. Lower levels were observed in patients with AD, VAD, and MIXED dementia, but not in those with other less common forms of dementia, compared with cognitively healthy subjects. The lowest activity was observed in patients with MCI, who exhibited the lowest levels of this enzymatic marker, followed, in decreasing order of effect magnitude, by patients with MIXED dementia, VAD, and AD.

However, unlike the results obtained in previous works for arylesterase activity, only MCI and MIXED dementia remained significantly lower than controls after multivariable adjustment, as well as in separate analyses stratified according to the main potential confounders, including age, sex, and HDL-C levels (although the differences involving MIXED dementia were more evident in subjects with the following features: women, younger and with high HDL-C). Therefore, based on the previous literature data we can hypothesize that the low levels of lactonase activity observed in MIXED dementia may be consistent with the combined presence of neurodegenerative and vascular pathological features. However, the present design did not allow us to demonstrate an additive effect of these processes. Nonetheless, this interpretation might be biologically plausible, given that both neurodegenerative and vascular mechanisms have been previously linked to oxidative stress and inflammation [50], two processes where PON1 in other studies demonstrated protective effects through its antioxidant and anti-inflammatory functions [51,52,53,54].

The present study adds to the current literature because it is among the very few investigating lactonase activity in peripheral fluids in AD and, to our knowledge, the only one evaluating this activity in MCI as well as in other major and minor types of dementia. The seminal study on this topic, which included a sample size comparable to ours of 204 AD patients and 230 controls, reported a slight but significant decrease in lactonase activity in AD compared with controls [30]. In that study, the authors reported that the difference remained significant after adjustment for covariates such as age, sex, and race, which were nearly the same covariates included in our study. Conversely, our adjusted model included also HDL-C, which was not considered in that study.

HDL-C levels, and even more importantly ApoA1 levels, represent key covariates in multivariate analyses involving PON1 activity. ApoA1 is the major apolipoprotein component of HDL [6,55], the lipoprotein that physically and functionally interacts with PON1 [56]. Since HDL is by far the main circulating carrier of PON1, changes in HDL concentration may inevitably influence the measured PON1 activity. Therefore, the lack of adjustment for HDL-related parameters may contribute to the partial discordance between studies. A further study by Bacchetti et al. reported a significant decrease in PON1 lactonase activity in AD compared with controls in a smaller sample of 83 participants [57]. Besides differences in covariate adjustment, other possible explanations for discrepancies across studies include methodological differences in the assessment of lactonase activity. For instance, Erlich et al., using 5-thiobutyl butyrolactone (TBBL) as a substrate, reported a stronger association between PON1 lactonase activity and AD than that observed in our study. However, their study population differed substantially from ours in terms of sample composition, which may partly account for the discrepancy between the findings [30].

A possible hypothesis to explain the findings of the present study concerns the relationship between peripheral and brain PON1.

Convincing evidence from preclinical studies has raised the possibility that circulating PON1, which is not substantially expressed in the brain, may be transferred to the central nervous system in ApoA1-containing particles [58]. However, the transport route and the relationship between circulating and cerebral PON1 activity remain to be clarified. Because the present study measured PON1 activity only in serum, it provides no direct evidence regarding blood–brain barrier transport or PON1 activity in the brain.

However, it can be hypothesized that PON1 levels and activity might be expected to show at least some degree of correspondence between the peripheral and central compartments.

In line with this hypothesis, in a previous study using the 3xTg-AD mouse model, we observed reduced brain PON1-related arylesterase activity, a reduction that was associated with a local impairment of redox homeostasis [27]. This observation supports the possibility that altered peripheral PON1 activity may, at least in part, reflect changes in brain redox regulation and PON1-related antioxidant defenses. However, based on our current results this hypothesis remains a mere speculation. In fact, whether circulating PON1 lactonase activity reflects cerebral PON1-related antioxidant defense in humans remains to be established.

Interestingly, even in this animal model, the early decrease in PON-related activity appears to be followed by stabilization at later stages. Therefore, based on previous works we can hypothesize that the PON-related impairment may represent an early event in AD-like pathology rather than a linearly progressive alteration, which is partly consistent with our observation. However, due to the cross-sectional nature of our study we cannot confirm the temporal sequence of changes in PON1 lactonase activity. Therefore, the lower activity observed in the MCI group and the higher activity observed in the AD group should be interpreted cautiously as possible evidence of an early decrease followed by stabilization within individuals.

Previous studies indicated that PON1 can limit oxidative modifications of LDL and HDL [59,60,61]. Based on this, reduced circulating lactonase activity could be hypothetically associated with diminished antioxidant protection of lipoproteins. However, in the present studies we did not measure HDL antioxidant function and therefore we cannot establish that lower lactonase activity caused HDL dysfunction or increased the level of lipoprotein oxidation. Nonetheless, in the present cohort we found an inverse association between PON1 lactonase activity and oxidized HDL only among controls, whereas no association was detected in the whole population or other patient groups. Despite this result, we are aware that this specific sub-group association does not establish a protective effect of PON1. Although this finding should be interpreted with caution because of the small subsample size, it is tempting to speculate that this preserved protective capacity may contribute to the association between higher lactonase activity and the absence of pathological hallmarks of AD and VAD, namely brain atrophy and leukoaraiosis, respectively, as evidenced in our study. Notably, this association was observed only in the control group.

In contrast, no association was found between lactonase activity and MPO, one of the main pro-oxidant enzymes that can enrich HDL and contribute to the shift from functional to dysfunctional lipoproteins [44,56,62,63], given that HDL oxidation has detrimental consequences for both the structural integrity and biological activity of this lipoprotein [52,64,65].

The present study has several limitations that should be acknowledged. First, its cross-sectional design prevents us from definitively establishing whether decreased PON1 lactonase activity is causally involved in MCI or MIXED dementia. Moreover, we cannot rule out the possibility that the observed reduction in PON1 activity is merely a consequence of the systemic oxidative stress that characterizes MCI and dementia, in line with in vitro evidence showing impaired enzymatic activity after exposure to high levels of reactive oxygen species. Longitudinal studies in cognitively healthy individuals are warranted to clarify this relationship and to determine whether reduced PON1 lactonase activity precedes cognitive decline. In contrast, our longitudinal data in MCI patients do not support a causal role of this activity in the progression from this prodromal condition to dementia. Second, the sizes of some diagnostic groups were limited, and this may have influenced at least part of the statistical analyses (e.g., correlation analysis). No a priori sample-size calculation was performed, and the statistical power was lower for the smaller VAD and other forms of dementia groups. As such, the absence of statistical significance in these analyses cannot exclude small to moderate associations which may have remained undetected. Third, unassessed confounding factors, such as anthropometric parameters, diet, hormonal status, and other lifestyle or metabolic variables, may have affected the validity and generalizability of the results. Fourth, the individuals enrolled in this study were not characterized by neuroimaging and CSF biomarkers of AD; therefore, misclassification of some participants cannot be ruled out. We are aware that the unavailability of this information limits the clinical relevance of our findings.

Fifth, the “Other forms of dementia” group included etiologically and clinically heterogeneous conditions, grouping both primary neurodegenerative dementias and other causes of cognitive impairment. These conditions were pooled to retain the full clinical spectrum of the recruited cohort, and to provide an exploratory comparison with the main diagnostic groups. Therefore, since this approach may have obscured diagnosis-specific differences in PON1 lactonase activity, the lack of a significant difference in this group should not be interpreted as evidence that PON1 lactonase activity is unaltered in each of its constituent conditions. Sixth, the lack of complete information on medication use, particularly statins, antioxidant supplements, and cholinesterase inhibitors, represents a limitation of the present study. To partially address this issue, we analyzed the available data on cholinesterase inhibitor use (n = 141) and antioxidant supplement use (n = 139). No significant differences in PON1 lactonase activity were observed between users and non-users of either treatment.

An additional limitation is the absence of information on PON1 polymorphisms in our cohort. The effects of common PON1 variants are substrate-dependent, with the most clearly established effects on paraoxonase activity [66]. However, the data on lactonase activity are still conflicting, with some studies reporting an influence of genotype on the activity when measured using dihydrocoumarin [23,24], and others demonstrating that lactonase activity is not influenced by any haplotype [67]. Because genotyping was not performed in the present cohort, we cannot exclude that differences in PON1 allele or haplotype distributions could have contributed to the observed differences between diagnostic groups. Future studies should integrate PON1 genotyping with measurements of lactonase activity as well as circulating PON1 concentration.

## 5. Conclusions

The main finding of the present study is that serum PON1 lactonase activity, one of the main contributors to the antioxidant properties of HDL, was significantly decreased in individuals with MCI, a condition considered as prodromal to dementia. A decreasing trend was also present in dementia-related disorders but appeared more evident in those forms characterized by a vascular component. However, the cross-sectional and associative nature of the study prevented us from drawing firm conclusions on the causal effect between this enzymatic activity and the pathological conditions investigated.

## Figures and Tables

**Table 1:** Main characteristics of the total sample.

	Controls (n = 109)	MCI (n = 139)	AD (n = 157)	VAD (n = 37)	MIXED Dementia (n = 199)	Other Forms of Dementia (n = 50)
Age (years)	76 (72–80)	79 (76–83) ^a^	79 (76–83) ^a^	81 (78–83) ^a^	81 (77–83) ^a^	78 (74–81) ^e^
Females (n, %)	68 (62)	78 (56)	111 (70)	26 (70)	132 (66)	29 (58)
Education (years)	8 (5–13)	5 (5–9)	5 (4–8) ^a,d^	5 (4–8)	5 (5–8) ^a^	5 (5–11)
Current smokers (n, %)	9 (8)	13 (9)	10 (6)	4 (11)	20 (10)	4 (8)
MMSE score	27 (26–29)	24 (22–26) ^a,b,c,d,e^	20 (18–22) ^a^	22 (18–24) ^a^	21 (18–24) ^a^	22 (18–25) ^a^
IADLs	7 (5–8)	5 (3–7)	4 (2–6) ^a^	5 (2–7) ^a^	4 (2–6) ^a^	5 (2–7)
BADLs	6 (5–6)	6 (5–6)	6 (5–6)	5 (4–6) ^a^	5 (5–6) ^a^	5 (4–6) ^a^
Hypertension (n, %)	68 (62)	97 (70)	107 (68)	30 (81)	138 (70)	29 (58)
Diabetes (n, %)	15 (14)	28 (20)	27 (17)	2 (5)	44 (22)	7 (14)
CHD (n, %)	8 (7)	20 (14)	15 (10)	4 (11)	28 (14)	5 (10)
Stroke (n, %)	4 (4)	5 (4)	3 (2)	2 (5)	13 (6)	2 (5)
Creatinine (mg/dL)	0.8 (0.7–1.0)	0.9 (0.8–1.0)	0.8 (0.7–1.0)	0.9 (0.8–1.2)	0.9 (0.7–1.2)	0.9 (0.7–1.0)
Albumin (g/dL)	4.1 (3.9–4.2)	4.0 (3.9–4.2)	4.0 (3.7–4.2) ^e^	3.9 (3.6–4.2)	3.9 (3.7–4.1) ^a^	4.0 (3.7–4.1)
Total Cholesterol (mg/dL)	206 ± 44	209 ± 45	210 ± 40	215 ± 33	201 ± 47	205 ± 40
HDL-C (mg/dL)	63 ± 18	62 ± 18	62 ± 15	59 ± 14	60 ± 18	59 ± 15
LDL-C (mg/dL)	123 ± 38	127 ± 38	128 ± 35	133 ± 26	120 ± 40	124 ± 33
Triglycerides (mg/dL)	99 (77–131)	100 (76–129)	93 (72–125)	102 (75–142)	98 (74–138)	87 (67–118)
Glucose (mg/dL)	94 (89–106)	97 (90–110)	93 (85–103)	95 (88–106)	95 (88–107)	96 (88–106)
Hemoglobin (g/dL)	13 (12–15)	13 (12–14)	13 (12–14)	14 (12–14)	13 (12–14)	13 (12–14)
Hs-CRP (mg/dL)	0.1 (0.1–0.3)	0.1 (0.1–0.3)	0.1 (0.1–0.3)	0.1 (0.1–0.2)	0.1 (0.1–0.3)	0.1 (0.0–0.3)

Mean ± SD for normally distributed variables; median (interquartile range) for not-normally distributed variables; and percentage for discrete variables. MMSE score Mini Mental State Examination score, CHD Coronary Heart Disease, HDL-C High-Density Lipoprotein-cholesterol, LDL-C Low-Density Lipoprotein-cholesterol, Hs-CRP High Sensitivity-C-Reactive Protein, IADL Instrumental Activities of Daily Living, and BADL Basic Activity of Daily Living. ^a^ *p* < 0.05 vs controls, ^b^ *p* < 0.05 vs MCI, ^c^ *p* < 0.05 vs AD, ^d^ *p* < 0.05 vs. VAD, and ^e^ *p* < 0.05 vs. MIXED dementia.

**Table 2:** Non-adjusted and adjusted means of PON1-lactonase activity across the study groups.

	Controls (n = 109)	MCI (n = 139)	AD (n = 157)	VAD (n = 37)	MIXED Dementia (n = 199)	Other Forms of Dementia (n = 50)
Non-adjusted means (95% CI)	3.6 ± 0.2 (3.2–3.9)	2.4 ± 0.1 ^a,c^ (2.1–2.6)	3.1 ± 0.1 ^a^ (2.8–3.2)	2.8 ± 0.2 ^a^ (2.4–3.1)	2.8 ± 0.1 ^a^ (2.6–3.0)	3.0 ± 0.2 (2.6–3.4)
Adjusted means ^#^ (95% CI)	3.4 ± 0.1 (3.1–3.7)	2.5 ± 0.1 ^a,d^ (2.1–2.7)	3.0 ± 0.1 (2.8–3.2)	2.8 ± 0.2 (2.4–3.3)	2.4 ± 0.1 ^a^ (2.6–3.0)	3.0 ± 0.1 (2.6–3.5)

Data are expressed as mean ± SEM. Unit of measurement: U/mL. ^#^ adjusting covariates: age, sex, hypertension, stroke, diabetes, smoking status, and HDL-C. *p* values (95% CI) for differences between non-adjusted means: ^a^ *p* < 0.05 vs. controls, ^c^ *p* < 0.05 vs. AD, ^d^ *p* < 0.05 vs. VAD.

## Data Availability

The raw data supporting the conclusions of this article will be made available by the authors on request.

## Supplementary Materials

The following supporting information can be downloaded at: https://www.mdpi.com/article/10.3390/antiox15081011/s1, Figure S1. Serum PON1 lactonase activity in MCI patients who converted to dementia and remained stable; Figure S2. Receiver operating characteristic (ROC) curves for PON1 lactonase activity for the diagnosis of MCI, AD, VAD, MIXED dementia; Figure S3. Comparison of Serum PON1 lactonase activity between women and men across the study sample stratified for diagnosis; Figure S4. Serum PON1 lactonase activity in women and men across the study samples; Figure S5. Serum PON1 lactonase activity across the study groups stratified by age (<79 vs. ≥79 years; median age of the overall sample); Figure S6. Serum PON1 lactonase activity across the study groups stratified by HDL-C levels (<59 vs. ≥59 mg/dL; median levels of the overall sample).

## Abbreviations

The following abbreviations are used in this manuscript:

AD	Alzheimer’s Disease
ApoA-I	Apolipoprotein A-I
BADL	Basic Activities of Daily Living
BSA	Bovine Serum Albumin
CDR	Clinical Dementia Rating
CHD	Coronary Heart Disease
DHC	Dihydrocoumarin
GDS	Geriatric Depression Scale
HDL-C	High-Density Lipoprotein Cholesterol
HRP	Horseradish Peroxidase
IADL	Instrumental Activities of Daily Living
LDL-C	Low-Density Lipoprotein Cholesterol
MCI	Mild Cognitive Impairment
MIXED dementia	MIXED Alzheimer’s Disease/Vascular Dementia
MMSE	Mini-Mental State Examination
MPO	Myeloperoxidase
MRI	Magnetic Resonance Imaging
NF-κB	Nuclear Factor kappa B
NIA-AA	National Institute on Aging–Alzheimer’s Association
NINDS-AIREN	National Institute of Neurological Disorders and Stroke–Association Internationale pour la Recherche et l’Enseignement en Neurosciences
NSAIDs	Nonsteroidal Anti-Inflammatory Drugs
oxHDL	Oxidized High-Density Lipoprotein
PON1	Paraoxonase-1
TBBL	5-Thiobutyl Butyrolactone
