# Supplementary material for: Reduced Serum PON1 Lactonase Activity Is Associated with Mild Cognitive Impairment and Mixed Dementia
Source: Antioxidants (Basel). 2026 Aug 13;15(8):1011. doi: 10.3390/antiox15081011 (PMC13509391; doi:10.3390/antiox15081011)
Supplement: Supplementary file 1 [file antioxidants-15-01011-s001.zip › antioxidants-4446412-supplementary.pdf]

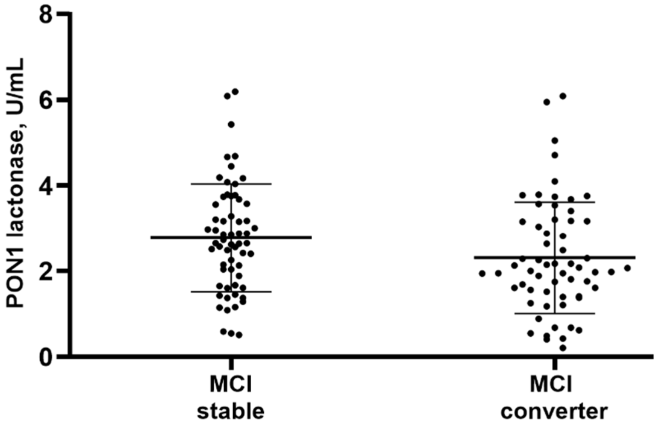

Figure S1. Serum PON1 lactonase activity in MCI patients who converted to dementia and remained stable

Serum PON1 lactonase was, although not significantly, higher in MCI stable compared to converter MCI

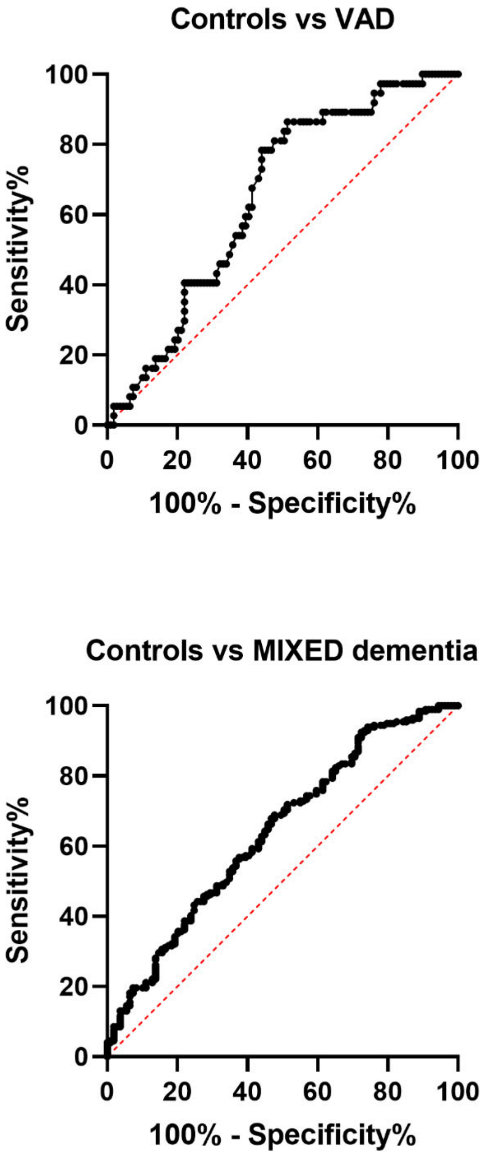

Figure S2. Receiver operating characteristic (ROC) curves for PON1 lactonase activity for the diagnosis of MCI, AD, VAD, MIXED dementia.

AUC values were 0.721 (sensitivity/specificity: 73/62%, cut-off = 2.5 U/mL) for MCI, 0.581 (sensitivity/specificity: 61/50%, cut off = 3.0 U/mL,) for AD, 0.641 (sensitivity/specificity: 61/60%, cut off = 2.9 U/mL) for VAD, 0.659 (sensitivity/specificity: 67/50%, cut off = 2.6 U/mL) for MIXED dementia

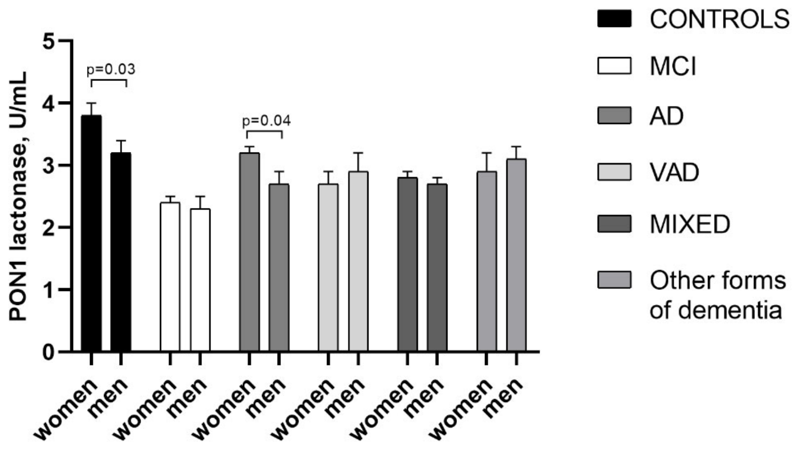

Figure S3. Comparison of Serum PON1 lactonase activity between women and men across the study sample stratified for diagnosis

Serum PON1 lactonase was significantly higher in women than in men only in CONTROLS and AD

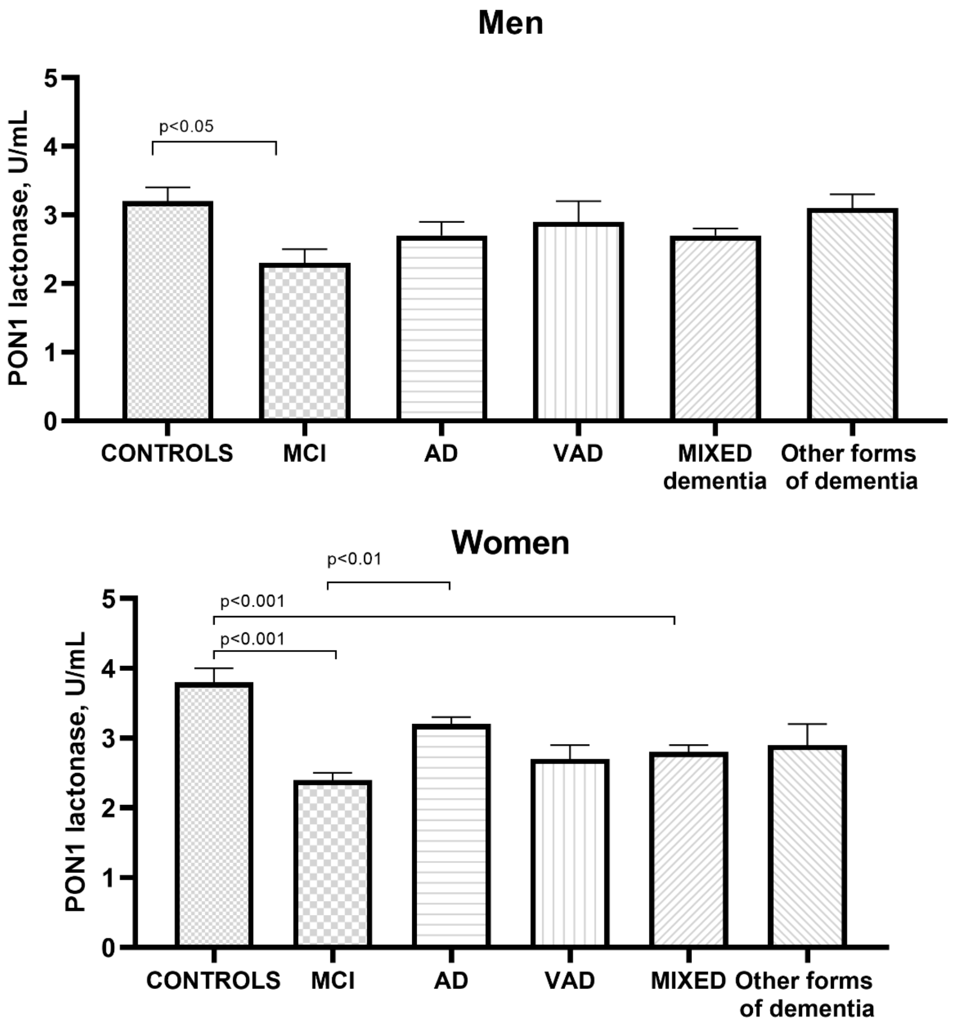

Figure S4. Serum PON1 lactonase activity in women and men across the study samples

Among men, serum PON1 lactonase activity differed significantly only between the CONTROL and MCI groups, with higher levels in CONTROLS. Among women, significant differences were observed between CONTROLS (higher) and both the MCI and MIXED groups, as well as between the MCI (lower) and AD groups.

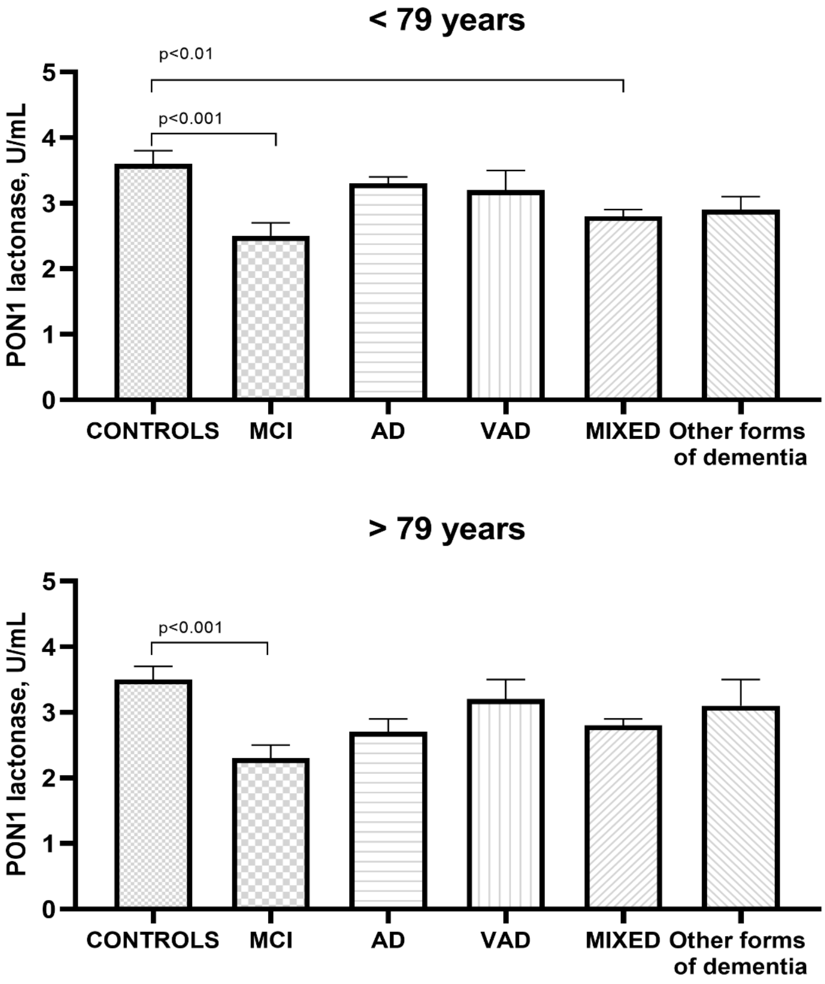

Figure S5. Serum PON1 lactonase activity across the study groups stratified by age (<79 vs. ≥79 years; median age of the overall sample).

In younger subjects, serum PON1 lactonase activity differed significantly between CONTROLS (higher) and both MCI and MIXED groups. Among older subject, a significant difference was observed between CONTROLS (higher) and MCI.

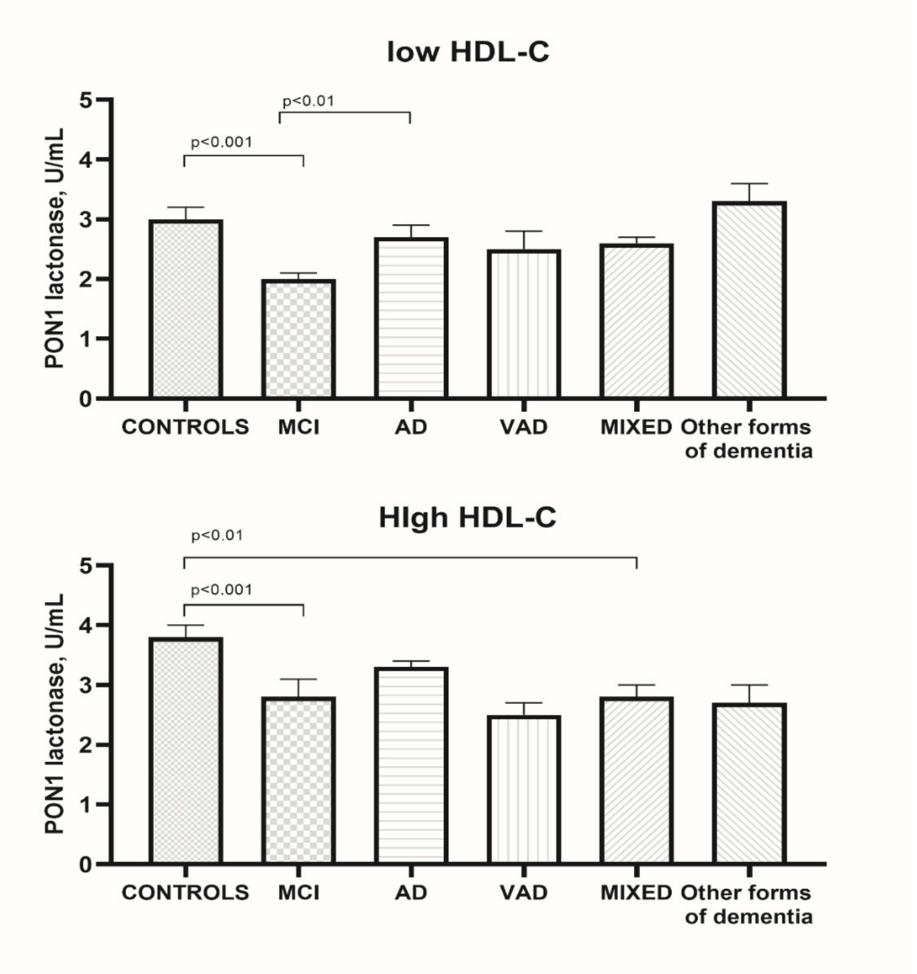

Figure S6. Serum PON1 lactonase activity across the study groups stratified by HDL-C levels (<59 vs. ≥59 mg/dL; median levels of the overall sample).

In subjects with lower HDL-C, serum PON1 lactonase activity differed significantly between CONTROLS (higher) and b MCI as well as between MCI (lower) and AD. Among subject, with high HDL-C CONTROLS (higher) differed from both MCI and MIXED
